# Development and validation of a metabolic syndrome-based nomogram to predict 1-year metastasis after radiotherapy in locally advanced nasopharyngeal carcinoma

**DOI:** 10.1080/07853890.2026.2743333

**Published:** 2026-10-11

**Authors:** Yanyu Chen, Hao Cheng, Changjun Xie, Ruimin He, Yaolin He, Haihui Xie, Aixin Li, Li Liang, Baiqi Wang, Yanhua Chen, Liqin Jiang, Yingqing Li, Ping Feng

**Affiliations:** a Department of Cancer Center, The Second Affiliated Hospital, Hengyang Medical School, University of South China, Hengyang, Hunan, China; b State Key Laboratory of Oncology in South China, Guangdong Key Laboratory of Nasopharyngeal Carcinoma Diagnosis and Therapy, Guangdong Provincial Clinical Research Center for Cancer, Sun Yat-sen University Cancer Center, Guangzhou, PR China

**Keywords:** Metabolic syndrome, nasopharyngeal carcinoma, nomogram

## Abstract

**Background:**

Metabolic syndrome (MetS) predicts outcomes in multiple malignancies. This study developed a MetS-integrated nomogram to estimate metastasis within one year after radiotherapy in locally advanced nasopharyngeal carcinoma (LA-NPC), extending existing models and facilitating personalized management.

**Methods:**

In this retrospective single-center study, 1333 LA-NPC patients undergoing radiotherapy-based multimodal therapy were randomly divided into training (*n* = 888) and validation (*n* = 445) cohorts. Univariate and multivariate logistic regression identified independent metastasis risk factors for the nomogram. Evaluation included C-index, calibration plots, probability density functions (PDFs), and clinical utility curves (CUCs). Discrimination was assessed using DeLong test for receiver operating characteristic (ROC) analysis.

**Results:**

Metastasis within one year occurred in 56 (6.3%) training and 28 (6.3%) validation patients. Pretreatment T stage, N stage, MetS, plasma EBV DNA, lactate dehydrogenase (LDH), C-reactive protein (CRP), and smoking were associated with metastasis; multivariate analysis confirmed MetS and EBV DNA as independent predictors. The nomogram incorporating T stage, N stage, MetS, and EBV DNA achieved C-indexes of 0.763 (95% CI: 0.694–0.832, training) and 0.715 (95% CI: 0.629–0.801, validation), surpassing single predictors (all *P* < 0.05). PDF and CUC analyses suggested an 8.7% threshold to optimize the identification of true positives and negatives, classifying 82.0% non-metastatic and 39.3% metastatic patients correctly.

**Conclusion:**

This MetS-based nomogram enables individualized risk stratification for metastasis within one year after radiotherapy in LA-NPC. The 8.7% threshold is exploratory but promising for identifying high-risk patients for further therapy or surveillance after external validation.

## Introduction

Nasopharyngeal carcinoma (NPC) represents a distinct epithelial malignancy of the head and neck with a pronounced geographical predilection, with over 70% of global incidence concentrated in East and Southeast Asia [1–3]. Its etiology is multifactorial, involving persistent Epstein-Barr virus (EBV) infection, genetic susceptibility, and environmental exposures [4,5]. Due to the lack of symptoms in the early stages, approximately 70% of patients are diagnosed at the locally advanced stage. Although current comprehensive treatment, including intensity-modulated radiotherapy (IMRT) combined with induction or concurrent chemotherapy, can increase the 5-year survival rate of locally advanced nasopharyngeal carcinoma to approximately 80%, approximately 15–20% of patients still experience distant metastasis within 1–3 years after radical treatment [2]. With the evolution of personalized treatment strategies, including the use of induction chemotherapy regimens such as TPF (docetaxel, cisplatin, and 5-fluorouracil) and GP (gemcitabine and cisplatin), as well as the incorporation of immunotherapy in the first-line setting, risk stratification has become increasingly important for guiding treatment intensification or de-escalation [6,7].

Current surveillance for distant metastasis predominantly depends on positron emission tomography-computed tomography (PET-CT). Despite PET-CT having high diagnostic sensitivity and specificity, its high cost makes it difficult to be used in every follow-up visit, resulting in some patients being unable to achieve early diagnosis. Therefore, it is still necessary to establish a more economical and more accurate prediction model to identify high-risk patients who are prone to distant metastasis.

Metabolic syndrome (MetS) represents a substantial public health concern, clinically characterized by the presence of at least three of the following components: central obesity, elevated fasting glucose, hypertension, and dyslipidemia. It is a well-recognized risk factor for various chronic conditions, including cardiovascular disease, type 2 diabetes mellitus, and chronic kidney disease [8–10]. An expanding body of epidemiological and clinical research further highlights a significant correlation between MetS and an increased risk for the development and progression of various cancers, thereby positioning MetS as a potential prognostic marker [11–15]. Existing prediction models for early metastasis in locally advanced nasopharyngeal carcinoma (LA-NPC) have primarily incorporated plasma EBV DNA, TNM staging, and various laboratory parameters. Recent studies have explored the utility of radiomics-based approaches using magnetic resonance imaging (MRI) [16,17] to enhance prediction accuracy. However, the specific prognostic utility of MetS, particularly in predicting distant metastasis within one year in LA-NPC, remains insufficiently investigated.

To address this gap, the present study aimed to develop and validate a comprehensive prognostic nomogram that incorporates MetS to predict the individualized risk of distant metastasis within one year following radiotherapy in patients with LA-NPC. This model is designed to facilitate risk stratification and inform tailored clinical management for high-risk patients.

## Methods and materials

### Patient cohort

We conducted a retrospective review of 1,333 consecutive patients diagnosed with locally advanced nasopharyngeal carcinoma (LA-NPC) who received radical radiotherapy at Sun Yat-sen University Cancer Center (Guangzhou, China) between April 2009 and October 2012. There were no missing data for the variables included in the final analysis, as the study inclusion criteria required complete clinical, laboratory, and follow-up data. Using computer-generated random numbers, patients were allocated in a 2:1 ratio into a training cohort (*n* = 888) for model development and an internal validation cohort (*n* = 445) for model assessment. Baseline demographic and clinical characteristics of both cohorts are presented in Table 1. The inclusion criteria were as follows: (1) histopathologically confirmed stage III–IV NPC according to the 8th edition of the American Joint Committee on Cancer (AJCC)/Union for International Cancer Control (UICC) staging system; (2) no evidence of distant metastasis at initial presentation, confirmed by baseline imaging; (3) no prior history of radiotherapy, chemotherapy, or other anticancer therapies; (4) absence of any other previous or concurrent malignancy; (5) availability of complete clinical, laboratory, and follow-up data for at least 12 months post-treatment. This study protocol was approved by the Institutional Review Board of Sun Yat-sen University Cancer Center. The requirement for written informed consent was waived due to the retrospective nature of the analysis.

**Table 1.** Patient demographics and clinical characteristics of the whole cohort*.

Characteristic	Training cohort (*n* = 888)	Validation cohort (*n* = 445)	*P* value
Gender			0.231
Female	207 (23.3%)	117 (26.3%)	
Male	681 (76.7%)	328 (73.7%)	
Age, years			0.400
<60	792 (89.2%)	390 (87.6%)	
≥60	96 (10.8%)	55 (12.4%)	
WHO type			0.300
I	6 (0.7%)	1 (0.2%)	
II	46 (5.2%)	17 (3.8%)	
III	836 (94.1%)	427 (96.0%)	
Tumor stage^a^			0.223
T1	40 (4.5%)	11 (2.5%)	
T2	52 (5.9%)	33 (7.4%)	
T3	565 (63.6%)	287 (64.5%)	
T4	231 (26.0%)	114 (25.6%)	
^a^Node stage			0.806
N0	90 (10.1%)	46 (10.3%)	
N1	472 (53.2%)	247 (55.5%)	
N2	196 (22.1%)	94 (21.1%)	
N3	130 (14.6%)	58 (13.0%)	
Metabolic syndrome			0.319
No	810 (91.2%)	413 (92.8%)	
Yes	78 (8.8%)	32 (7.2%)	
EBV DNA, copies/ml			0.931
<1000	564 (63.5%)	286 (64.3%)	
1000–9999	236 (26.6%)	114 (25.6%)	
≥10000	88 (9.9%)	45 (10.0%)	
Body mass index, kg/m²			0.510
<18.5	67 (7.5%)	41 (9.2%)	
18.5–24.9	599 (67.5%)	303 (68.1%)	
25.0–29.9	198 (22.3%)	93 (20.9%)	
≥30.0	24 (2.7%)	8 (1.8%)	
Smoking			0.082
No	531 (59.8%)	288 (64.7%)	
Yes	357 (40.2%)	157 (35.3%)	
Drinking			0.361
No	780 (87.8%)	383 (86.1%)	
Yes	108 (12.2%)	62 (13.9%)	
Family history of cancer			0.615
No	656 (73.9%)	323 (72.6%)	
Yes	232 (26.1%)	122 (27.4%)	
ALT, U/L			0.828
≤50	809 (91.1%)	407 (91.5%)	
>50	79 (8.9%)	38 (8.5%)	
AST, U/L			0.544
≤40	845 (95.2%)	420 (94.4%)	
>40	43 (4.8%)	25 (5.6%)	
LDH, U/L			0.244
≤250	835 (94.0%)	411 (92.4%)	
>250	53 (6.0%)	34 (7.6%)	
CRP, mg/L			0.186
≤3	560 (63.1%)	297 (66.7%)	
>3	328 (36.9%)	148 (33.3%)	
Metastasis within one year			0.992
No	832 (93.7%)	417 (93.7%)	
Yes	56 (6.3%)	28 (6.3%)	

*Pearson χ^2^ test was used to calculate the *P* value.

WHO, World Health Organization; EBV DNA = Epstein–Barr virus DNA; CRP = C-reactive protein; LDH = serum lactate dehydrogenase levels; NPC = nasopharyngeal carcinoma; ALT = alanine aminotransferase; AST = aspartate aminotransferase.

^a^According to the eighth edition of UICC/AJCC staging system.

### Baseline assessment and diagnosis

Before initiating treatment, all patients underwent a comprehensive clinical evaluation, including a detailed medical history, physical examination, and documentation of age, sex, height, weight, body mass index (BMI), family history of NPC, and smoking status. Peripheral venous blood samples were obtained for laboratory analyses, including quantification of plasma Epstein-Barr virus (EBV) DNA, C-reactive protein (CRP), lactate dehydrogenase (LDH), alanine aminotransferase (ALT) and aspartate aminotransferase (AST). Diagnostic imaging comprised contrast-enhanced magnetic resonance imaging (MRI) of the head and neck to delineate the primary tumor extent and regional lymph node involvement. Staging for distant metastasis was performed using a combination of 18 F-fluorodeoxyglucose positron emission tomography/computed tomography (PET/CT), contrast-enhanced chest CT, abdominal ultrasonography or contrast-enhanced abdominal CT, and whole-body bone scintigraphy, as clinically indicated.

### Treatment protocols

Treatment was administered in accordance with contemporary institutional guidelines for LA-NPC. All patients received radiotherapy as the primary local modality. The radiotherapy regimen involved intensity-modulated radiotherapy (IMRT) with a prescribed dose of 66–72 Gy in 28–33 fractions to the primary gross tumor volume (PGTVnx) and 64–70 Gy in 28–33 fractions to involved cervical lymph nodes (PGTVnd), delivered five days per week. The majority of patients (*n* = 1262, 94.7%) received concurrent platinum-based chemotherapy, while a minority (*n* = 71, 5.3%) received radiotherapy alone due to contraindications to chemotherapy or patient choice. The specific chemotherapy regimen (concurrent with or without induction) was selected based on multidisciplinary team assessment.

### Quantification of plasma EBV DNA

Plasma EBV DNA load was measured from pre-treatment blood samples using a real-time quantitative polymerase chain reaction (qPCR) assay. The assay targeted the conserved *BamHI*-W region of the EBV genome. The primer sequences were: forward, 5′-AGT CTC TGC CTC AGG GCA-3′ (W-44F); reverse, 5′-ACA GAG GGC CTG CTG TCC ACC G-3′ (W119R). Amplification was detected using a dual-labeled fluorescent TaqMan probe: 5′-[FAM]CAC TGT CTG TAA AGT CCA GCC TCC[TAMRA]-3′ (W-67T). Results were expressed as copies per milliliter.

### Definition of metabolic syndrome

Metabolic syndrome (MetS) was diagnosed according to the 2004 criteria established by the Chinese Diabetes Society. Diagnosis required the presence of at least three of the following four components:

1. **Obesity:** Body mass index (BMI) ≥ 25 kg/m^2^.
2. **Hyperglycemia:** Fasting plasma glucose ≥ 6.1 mmol/L and/or 2-hour postprandial glucose ≥ 7.8 mmol/L, or a previous diagnosis of diabetes mellitus under treatment.
3. **Hypertension:** Systolic blood pressure ≥ 140 mmHg and/or diastolic blood pressure ≥ 90 mmHg, or current use of antihypertensive medication.
4. **Dyslipidemia:** Serum triglycerides ≥ 1.7 mmol/L and/or high-density lipoprotein (HDL) cholesterol < 1.0 mmol/L in men or < 1.3 mmol/L in women.

### Follow-up and study endpoint

Post-treatment surveillance was conducted according to a standardized protocol. Patients were assessed every 3 months during the first year, and annually thereafter. Follow-up evaluations included physical examination, nasopharyngoscopy, plasma EBV DNA testing, chest radiography, abdominal ultrasonography, and neck MRI or CT. If clinical symptoms or routine tests suggested recurrence or metastasis, confirmatory imaging (e.g. PET/CT, contrast-enhanced MRI/CT) or biopsy was performed. The primary endpoint was distant metastasis within 12 months post-radiotherapy; absence of metastasis was confirmed by imaging and/or histopathology.

### Statistical analysis

Statistical analyses were performed using SPSS (version 26.0, IBM Corp.), MedCalc Statistical Software (version 18.3), and R software (version 4.0.0; R Foundation for Statistical Computing). Multicollinearity among candidate predictors was assessed using variance inflation factor (VIF), with a threshold of VIF > 5 indicating significant collinearity. Continuous variables were categorized based on established clinical cut-off values or optimal thresholds determined by receiver operating characteristic (ROC) curve analysis. Plasma EBV DNA levels were stratified into three categories (<1000,1000–9999,≥10000 copies/mL) based on clinical relevance and previous literature [18]. Univariate analyses were first performed to identify potential predictors of metastasis within one year. Variables with a *P* value < 0.05 in univariate analyses were subsequently entered into a multivariate logistic regression model to identify independent prognostic factors. Variables were selected for inclusion in the final model based on the following criteria: (1) statistical significance in multivariate analysis (*P* < 0.05), and (2) established clinical importance supported by prior literature. T stage and N stage were included in the final nomogram irrespective of their statistical significance in multivariate analysis because both are universally recognized as fundamental prognostic determinants in NPC and are routinely used in clinical decision-making [19–21]. The discriminative ability of the nomogram was evaluated using Harrell’s concordance index (C-index). Calibration was assessed visually with calibration plots. The predictive performance of the nomogram was compared against individual clinical predictors using ROC curve analysis and the DeLong test. To inform clinical application, probability density function (PDF) plots were generated to visualize the distribution of predicted probabilities between patients with and without metastasis. Greater separation between the two density curves indicates better discriminative performance, whereas overlapping regions highlight diagnostic uncertainty. Clinical utility curves (CUCs) were employed to evaluate the net benefit of using the nomogram across a range of threshold probabilities. In these curves, net benefit is calculated as true positives minus false positives weighted by the odds of the threshold, thereby quantifying the benefit-harm trade-off at each decision threshold. This combined approach enabled the identification of an optimal cutoff that maximizes net benefit while maintaining an acceptable balance between sensitivity and specificity.

## Results

### Patient characteristics

The study enrollment flow is illustrated in Figure 1. The baseline demographic and clinicopathological characteristics of the 888 patients in the training cohort and the 445 patients in the validation cohort are summarized in Table 1. The median age was 45 years in the training cohort and 46 years in the validation cohort. According to the 8th edition of the AJCC/UICC staging system, the distribution of T categories in the training cohort was as follows: T1, 40 patients (4.5%); T2, 52 (5.9%); T3, 565 (63.6%); and T4, 231 (26.0%). In the validation cohort, the distribution was T1 in 11 patients (2.5%), T2 in 33 (7.4%), T3 in 287 (64.5%), and T4 in 114 (25.6%). Regarding N categories, the training cohort consisted of N0 in 90 patients (10.1%), N1 in 472 (53.2%), N2 in 196 (22.1%), and N3 in 130 (14.6%). Corresponding figures for the validation cohort were N0 in 46 (10.3%), N1 in 247 (55.5%), N2 in 94 (21.1%), and N3 in 58 (13.0%).

**Figure 1.**
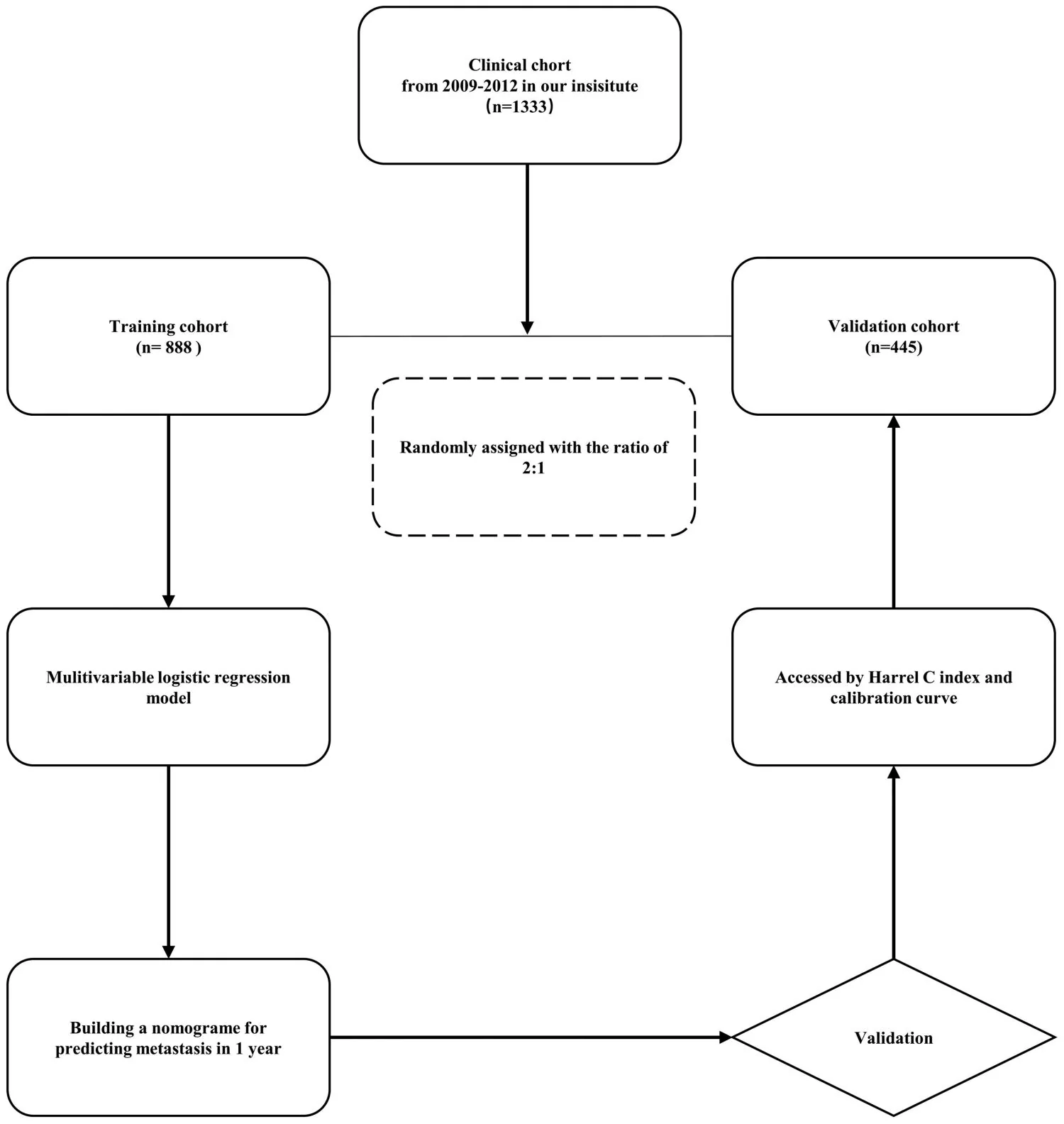
Developing and validating a program for predicting metastasis within one year in nasopharyngeal carcinoma.

The median follow-up duration was 95.1 months in the training cohort and 95.5 months in the validation cohort. The primary endpoint of distant metastasis within one-year post-radiotherapy was observed in 56 patients (6.3%) in the training cohort and 28 patients (6.3%) in the validation cohort.

### Univariate analyses of predictors for metastasis within one year

Associations between pretreatment variables and the development of metastasis within one year were assessed using univariate analyses (Table 2). In the training cohort, T category, N category, metabolic syndrome (MetS), plasma EBV DNA level, and serum LDH level were significantly associated with an increased risk of metastasis within one year (all *P* < 0.05). In the validation cohort, significant associations were found for smoking history and serum CRP level. No other variables examined demonstrated a significant correlation with metastasis within one year in the univariate analyses.

**Table 2.** Patient demographics and clinical characteristics of training cohort and validation cohort, respectively*.

	Training cohort (*n* = 888)		*P* value	Validation cohort (*n* = 445)		*P* value
Characteristic	No Metastasis within one year (*n* = 832)	Metastasis within one year (*n* = 56)		No Metastasis within one year (*n* = 417)	Metastasis within one year (*n* = 28)	
**Gender**			0.502			0.546
Female	196 (23.6%)	11 (19.6%)		111 (26.6%)	6 (21.4%)	
Male	636 (76.4%)	45 (80.4%)		306 (73.4%)	22 (78.6%)	
**Age, y**			0.387			0.981
<60	744 (89.4%)	48 (85.7%)		366 (87.8%)	24 (85.7%)	
≥60	88 (10.6%)	8 (14.3%)		51 (12.2%)	4 (14.3%)	
**WHOtype**			0.692			0.141
I	6 (0.7%)	0 (0%)		1 (0.2%)	0 (0%)	
II	44 (5.3%)	2 (3.6%)		14 (3.4%)	3 (10.7%)	
III	782 (94.0%)	54 (96.4%)		402 (96.4%)	25 (89.3%)	
**Tumor stage** ^a^			0.003			0.306
T1	37 (4.4%)	3 (5.4%)		10 (2.4%)	1 (3.6%)	
T2	47 (5.6%)	5 (8.9%)		33 (7.9%)	0 (0%)	
T3	542 (65.1%)	23 (41.1%)		270 (64.7%)	17 (60.7%)	
T4	206 (24.8%)	25 (44.6%)		104 (24.9%)	10 (35.7%)	
**Node stage** ^a^			<0.001			0.130
N0	86 (10.3%)	4 (7.1%)		46 (11.0%)	0 (0%)	
N1	456 (54.8%)	16 (28.6%)		233 (55.9%)	14 (50.0%)	
N2	180 (21.6%)	16 (28.6%)		86 (20.6%)	8 (28.6%)	
N3	110 (13.2%)	20 (35.7%)		52 (12.5%)	6 (21.4%)	
**Metabolic syndrome**			0.047			1.000
No	763 (91.7%)	47 (83.9%)		387 (92.8%)	26 (92.9%)	
Yes	69 (8.3%)	9 (16.1%)		30 (7.2%)	2 (7.1%)	
**EBV DNA, copies/ml**			<0.001			0.749
<1000	544 (65.4%)	20 (35.7%)		269 (64.5%)	17 (60.7%)	
1000–9999	217 (26.1%)	19 (33.9%)		107 (25.7%)	7 (25.0%)	
≥10000	71 (8.6%)	17 (30.4%)		41 (9.8%)	4 (14.3%)	
**Body mass index, kg/m²**			0.459			0.602
<18.5	62 (7.5%)	5 (8.9%)		40 (9.6%)	1 (3.6%)	
18.5–24.9	558 (67.1%)	41 (73.2%)		283 (67.9%)	20 (71.4%)	
25.0–29.9	188 (22.6%)	10 (17.9%)		86 (20.6%)	7 (25.0%)	
≥30.0	24 (2.9%)	0 (0%)		8 (1.9%)	0 (0%)	
**Smoking**			0.326			0.036
No	501 (60.2%)	30 (53.6%)		275 (65.9%)	13 (46.4%)	
Yes	331 (39.8%)	26 (46.4%)		142 (34.1%)	15 (53.6%)	
**Drinking**			0.444			0.237
No	729 (87.6%)	51 (91.1%)		361 (86.6%)	22 (78.6%)	
Yes	103 (12.4%)	5 (8.9%)		56 (13.4%)	6 (21.4%)	
**Family history of Cancer**			0.146			0.562
No	610 (73.3%)	46 (82.1%)		304 (72.9%)	19 (67.9%)	
Yes	222 (26.7%)	10 (17.9%)		113 (27.1%)	9 (32.1%)	
**ALT, U/L**			1.000			0.534
≤50	758 (91.1%)	51 (91.1%)		380 (91.1%)	27 (96.4%)	
>50	74 (8.9%)	5 (8.9%)		37 (8.9%)	1 (3.6%)	
**AST, U/L**			0.871			0.951
≤40	791 (95.1%)	54 (96.4%)		393 (94.2%)	27 (96.4%)	
>40	41 (4.9%)	2 (3.6%)		24 (5.8%)	1 (3.6%)	
**LDH, U/L**			0.007			0.083
≤250	787 (94.6%)	48 (85.7%)		388 (93.0%)	23 (82.1%)	
>250	45 (5.4%)	8 (14.3%)		29 (7.0%)	5 (17.9%)	
**CRP, mg/L**			0.343			0.018
≤3	528 (63.5%)	32 (57.1%)		284 (68.1%)	13 (46.4%)	
>3	304 (36.5%)	24 (42.9%)		133 (31.9%)	15 (53.6%)	

*Pearson χ^2^ test was used to calculate the *P* value.

WHO, World Health Organization; EBV DNA = Epstein–Barr virus DNA; CRP = C-reactive protein; LDH = serum lactate dehydrogenase levels; NPC = nasopharyngeal carcinoma; ALT = Alanine Aminotransferase; AST = Aspartate Aminotransferase.

^a^According to the eighth edition of UICC/AJCC staging system.

### Multivariate analysis identifying independent prognostic factors

We considered all potential prognostic factors related to clinical features and laboratory indicators, including T stage, N stage, metabolic syndrome (MetS), EBV DNA, LDH, CRP, and smoking history. The results of the multivariate logistic analysis are presented in Table 3. According to the findings of this study, both MetS and EBV DNA were independent prognostic factors for metastasis within one year. For MetS, we found that patients with MetS had a higher risk of metastasis within one year (OR = 2.285, 95% CI = 1.018–5.130, *P* = 0.045). Compared with patients with EBV DNA <1000 copies/mL, the ORs were 1.815 for 1000–9999 copies/mL and 4.441 for ≥10000 copies/mL (*P* < 0.001). Additionally, laboratory indicators such as LDH (≥250 U/L) and CRP (≥3 mg/L), as well as smoking history, were not proven to be independent risk factors (LDH: OR = 1.301, 95% CI = 0.531–3.190, *P* = 0.565; CRP: OR = 0.806, 95% CI = 0.441–1.474, *P* = 0.484; Smoking: OR = 1.305, 95% CI = 0.737–2.313, *P* = 0.361).

**Table 3.** Multivariable analysis of the training cohort*.

	Multivariate analysis		Selected factors for building the model	
	OR (95% CI)	*P* value	OR (95% CI)	*P* value
Tumor stage^a^				
T1	Reference		Reference	
T2	0.922 (0.191, 4.437)	0.919	0.979 (0.205, 4.659)	0.979
T3	0.940 (0.253, 3.502)	0.927	0.947 (0.256, 3.508)	0.935
T4	2.706 (0.709, 10.326)	0.145	2.707 (0.718, 10.203)	0.141
Node stage^a^				
N0	Reference		Reference	
N1	0.621 (0.196, 1.967)	0.418	0.625 (0.197, 1.983)	0.425
N2	1.612 (0.495, 5.246)	0.428	1.595 (0.490, 5.200)	0.438
N3	2.830 (0.837, 9.566)	0.094	2.808 (0.831, 9.485)	0.096
Metabolic syndrome				
No	Reference		Reference	
Yes	2.285 (1.018, 5.130)	0.045	2.230 (1.003, 4.958)	0.049
EBV DNA, copies/ml				
<1000	Reference		Reference	
1000–9999	1.815 (0.920, 3.580)	0.085	1.809 (0.921, 3.555)	0.085
≥10000	4.441 (2.040, 9.668)	<0.001	4.311 (2.029, 9.158)	<0.001
LDH, U/L				
≤250	Reference			
>250	1.301 (0.531, 3.190)	0.565		
CRP, mg/L				
≤3	Reference			
>3	0.806 (0.441, 1.474)	0.484		
Smoking				
No	Reference			
Yes	1.305 (0.737, 2.313)	0.361		

*Odds ratios, 95% confidence intervals, and *P* values were estimated using logistic regression with the Wald method. All statistical tests were two-sided.

CI = confidence interval; OR = odds ratio; EBV DNA = Epstein–Barr virus DNA; LDH = serum lactate dehydrogenase levels; CRP = C-reactive protein; NPC = nasopharyngeal carcinoma.

^a^According to the eighth edition of UICC/AJCC staging system.

### Development and internal validation of the prognostic nomogram

A prognostic nomogram was constructed to provide an individualized prediction of the probability of metastasis within one year (Figure 2A). The model incorporated the two independent factors identified in the multivariate analysis (MetS and EBV DNA) and included T and N categories due to their established clinical relevance, aiming to enhance the model’s clinical utility and face validity [19–21]. Collinearity diagnostics using variance inflation factor (VIF) were performed for all four variables (T, N, MetS, and EBV DNA), and all VIF values were below 5, indicating no significant multicollinearity. Points are assigned for each variable level, and the total points correspond to a predicted probability on the bottom scale. Internal validation used 1,000 bootstrap iterations with patient-level resampling with replacement from the training cohort (*n* = 888). C-index was 0.763 (95% CI 0.694–0.832) in training cohort and 0.715 (95% CI 0.629–0.801) in validation cohort. The validation cohort was not used for fitting or tuning. Calibration plots (Figure 2B) showed good agreement between the nomogram-predicted probabilities and the actual observed outcomes in both cohorts. The Brier scores were 0.054 for the training cohort and 0.057 for the validation cohort, indicating good calibration. The detailed point assignment for each variable in the nomogram is provided in Table 4.

**Figure 2.**
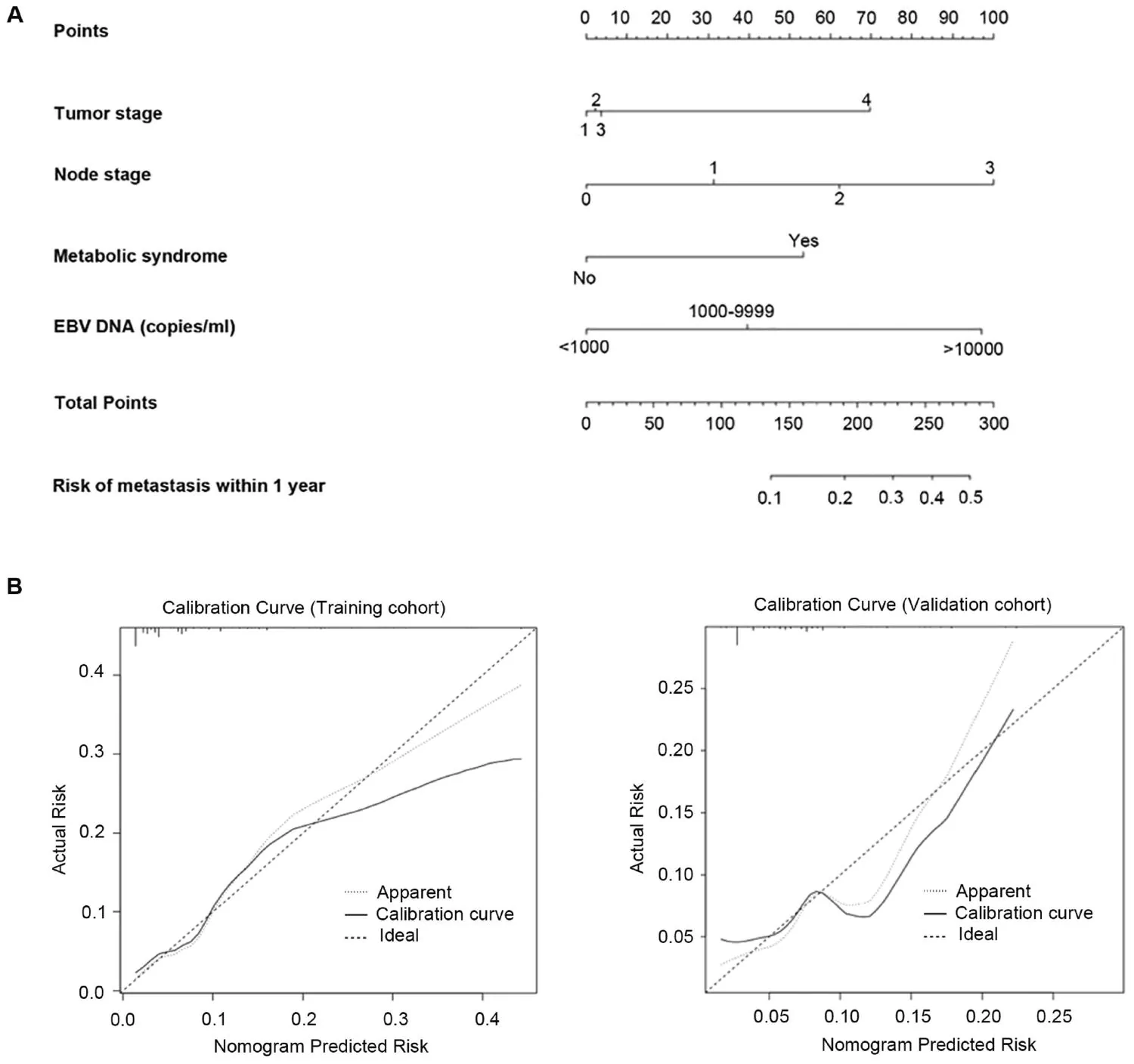
Nomogram, including pretreatment tumor stage, node stage, metabolic syndrome, and EBV DNA, for predicting metastasis within 1 year after radiotherapy in patients with locoregionally advanced nasopharyngeal carcinoma(LANPC). This nomogram allows the users to obtain the probability of risk of metastasis within 1 year corresponding to patients’ combining of covariates (A); calibration plots for the prediction nomogram in the training and validation cohorts (B). EBV DNA = Epstein–Barr virus DNA.

**Table 4.** Nomogram score of each factor in model.

Variate	Nomogram score
Tumor stage^a^	
T1	0
T2	2
T3	4
T4	70
Node stage^a^	
N0	0
N1	31
N2	62
N3	100
Metabolic syndrome	
No	0
Yes	53
EBV DNA, copies/ml	
<1000	0
1000–9999	39
≥10000	97

EBV DNA = Epstein–Barr virus DNA.

^a^According to the eighth edition of UICC/AJCC staging system.

### Comparative performance and clinical application of the nomogram

The concordance index (C-index) for all variables included in the nomogram is presented in Table 5, and the ROC curves comparing each individual factor with the nomogram are shown in Figure 3. The DeLong test for ROC curve analysis was used to compare the predictive ability for distant metastasis within one year in patients with locally advanced nasopharyngeal carcinoma. The nomogram demonstrated a significantly superior C-index compared to any single independent factor. Comparisons of the C-index between the nomogram and other factors are as follows: in the training cohort, nomogram vs. T category (0.763 vs. 0.626, *P* < 0.001), nomogram vs. N category (0.763 vs. 0.672, *P* = 0.003), nomogram vs. metabolic syndrome (MetS) (0.763 vs. 0.539, *P* < 0.001), and nomogram vs. EBV DNA (0.763 vs. 0.673, *P* = 0.003). In the validation cohort, the comparisons were: nomogram vs. T category (0.715 vs. 0.584, *P* = 0.017), nomogram vs. N category (0.715 vs. 0.616, *P* < 0.001), nomogram vs. MetS (0.715 vs. 0.500, *P* < 0.001), and nomogram vs. EBV DNA (0.715 vs. 0.525, *P* = 0.003).

**Figure 3.**
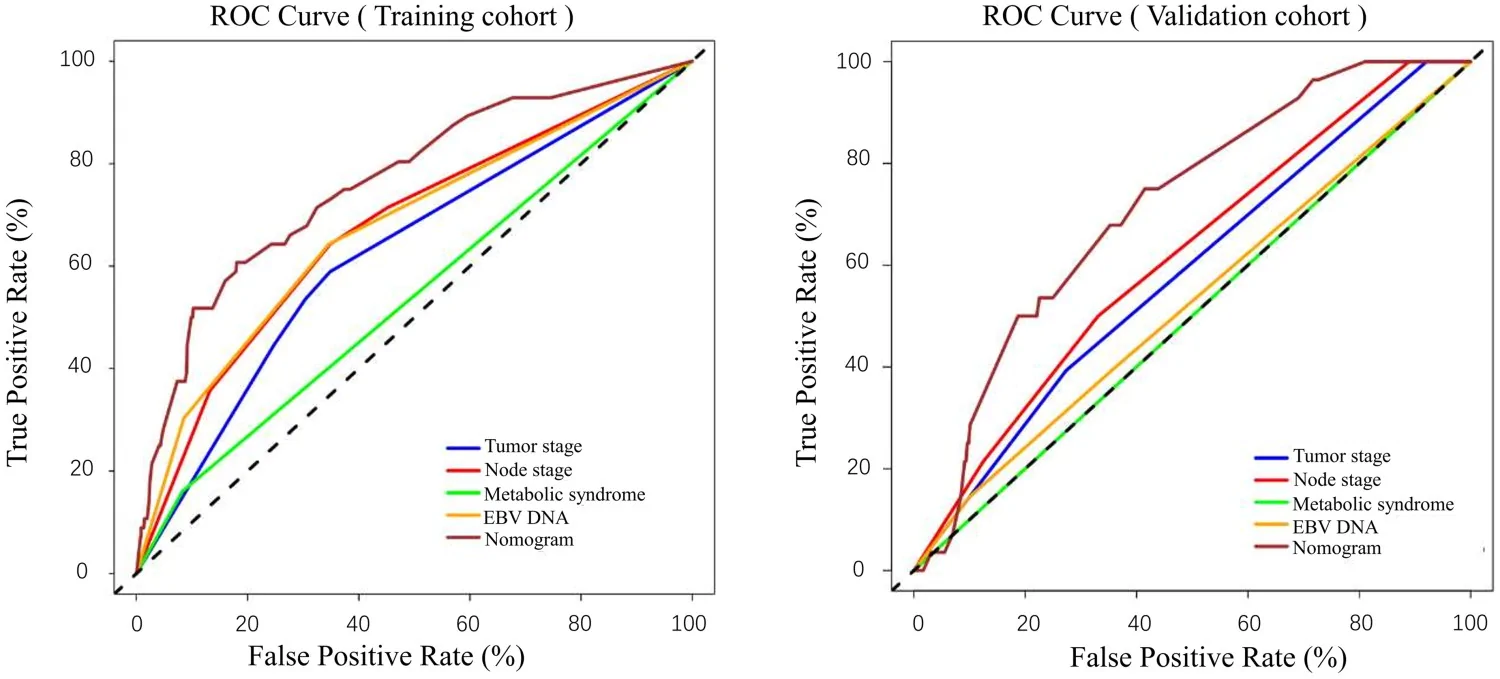
Receiver-operating characteristic (ROC) curve comparing the accuracy of nomogram with Tumor stage, node stage, metabolic syndrome, and pretreatment EBV DNA for predicting metastasis within one year. EBV DNA = Epstein–Barr virus DNA; C-index = concordance index.

**Table 5.** The C-index of nomogram, T, N,MS, and EBV DNA for prediction of metastasis within one year in the training cohort and validation cohort*.

Factor	Training cohort		Validation cohort	
	C index (95% CI)	*P* value	C index (95% CI)	*P* value
Nomogram	0.763 (0.694, 0.832)		0.715 (0.629, 0.801)	
Tumor stage	0.626 (0.556, 0.696)		0.584 (0.495, 0.673)	
Node stage	0.672 (0.599, 0.745)		0.616 (0.524, 0.708)	
Metabolic syndrome	0.539 (0.489, 0.588)		0.500 (0.450, 0.550)	
EBV DNA	0.673 (0.601, 0.746)		0.525 (0.425, 0.625)	
Nomogram vs. tumor stage		<0.001		0.017
Nomogram vs. node stage		0.003		<0.001
Nomogram vs. metabolic syndrome		<0.001		<0.001
Nomogram vs. EBV DNA		0.003		0.003

*Nomogram: including four risk factors (tumor stage, node stage, metabolic syndrome, and plasma EBV DNA). Delong test were used for comparing C-index.

CI = confidence interval; EBV DNA = Epstein–Barr virus DNA; C-index = concordance index.

To define a clinically applicable risk threshold, probability density function (PDF) and clinical utility curves (CUCs) were generated in the training cohort. The PDF (Figure 4A) for patients without metastasis within one year displayed a sharp peak concentrated in the low-risk range (0%–8.7%), whereas that for patients with metastasis exhibited a flatter, right-skewed distribution with overlapping regions. The CUC (Figure 4B) quantified the net clinical benefit across a range of threshold probabilities. Balancing the identification of true positives and true negatives, an exploratory threshold of 8.7% was selected and showed promising ability to distinguish patients with and without metastasis within one year. At this cutoff, the model correctly identified 82.0% of patients without metastasis and 39.3% of those with metastasis within one year in the training cohort. These findings support further evaluation of this threshold in external and prospective studies.

**Figure 4.**
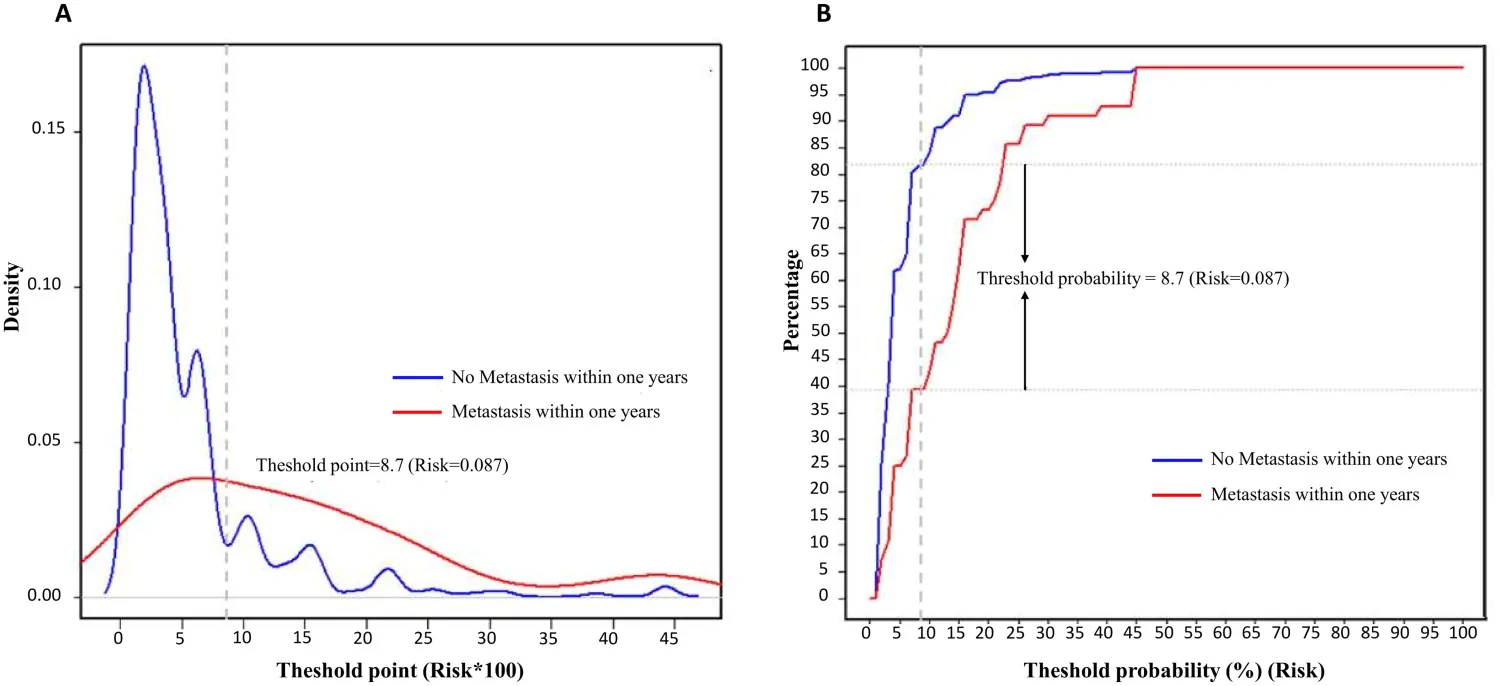
The probability density function plot (PDF) (A) and clinical utility curve plot (CUC) (B) to compare and choose the optimal thresholds.

## Discussion

The development of distant metastasis within one year after radiotherapy in patients with locally advanced nasopharyngeal carcinoma (LA-NPC) is associated with an extremely poor prognosis and can significantly undermine patients’ confidence and adherence to subsequent treatments. Therefore, developing accurate predictive tools for this patient population is of paramount importance. In this study, we selected a cohort of 1,333 patients with LA-NPC to construct a prognostic nomogram. To our knowledge, this is the first nomogram specifically incorporating metabolic syndrome (MetS) as a predictor for metastasis within one year after radiotherapy in LA-NPC patients.

In this study, both clinical factors and laboratory parameters were analyzed. We identified MetS and plasma EBV DNA level as independent predictors for metastasis within one year after radiotherapy in LA-NPC patients. T category and N category have been well established as independent prognostic factors in NPC in prior studies [19–21]; consequently, these variables were also included in the nomogram.

Our findings demonstrate that MetS is an independent predictor for metastasis within one year in LA-NPC patients. MetS, comprising hypertension, hyperglycemia, and dyslipidemia, is widely recognized as a significant risk factor for cardiovascular disease [22]. Accumulating evidence suggests that MetS is also associated with an increased risk of tumorigenesis and can serve as an indicator of poor prognosis for various cancers. The biological basis linking MetS to metastasis involves chronic inflammation, insulin resistance, and immune dysregulation [21–24]. In NPC, it has been reported that patients with concurrent MetS have a worse clinical outcome, potentially mediated by metabolic pathways involving d-glutamine and d-glutamate metabolism, as well as the biosynthesis of valine, leucine, and isoleucine [21]. Furthermore, distinct lipoprotein profiles have been linked to the prognosis of NPC patients [25–30]. Huang et al. [31] demonstrated that pretreatment BMI is a simple and reliable independent prognostic factor in LA-NPC patients. In their study, patients with a BMI <18.5 kg/m^2^ had the lowest 5-year failure-free survival rate, which may be explained by malnutrition potentially reducing a patient’s ability to tolerate tumor treatment [32,33]. A meta-analysis by Guo et al. [34] concluded that diabetes did not affect the prognosis of NPC patients. However, Peng et al. [35] reported shorter survival in diabetic patients compared to non-diabetic patients. Yang et al. [36] found that hypertension was significantly associated with an unfavorable prognosis in NPC. Overall, the prognostic value of individual components of MetS remains somewhat controversial. Importantly, no large-scale studies have evaluated the predictive value of the full MetS constellation for metastasis within one year after radiotherapy in LA-NPC patients.

On the other hand, our study also confirms that plasma EBV DNA level is a significant predictor for metastasis in LA-NPC patients. Studies by Tang et al. [37] and Lo et al. [38] have established EBV DNA as a crucial prognostic indicator in NPC, where tenfold differences in plasma levels have proven to be an excellent tool for predicting overall survival and disease progression. The significant predictive value of EBV DNA for metastasis within six months post-radiotherapy has also been corroborated by Lu et al. [18]. Therefore, our findings are consistent with the established literature.

The nomogram developed in this study showed promising discriminative ability and calibration, with C-indices of 0.763 and 0.715 in the training and validation cohorts, respectively. Comparative ROC analysis confirmed that the model’s predictive performance was superior to that of any individual risk factor. This scoring system may help clinicians estimate the individualized risk of metastasis within one year in LA-NPC patients, thereby supporting risk stratification and potentially informing personalized management in future validated studies.

In this study, a threshold probability of 8.7% was selected as an exploratory cutoff for risk stratification, based on balancing the proportions of true positives and true negatives. At this cutoff, the model correctly identified approximately 82.0% of patients who did not develop metastasis and approximately 39.3% of those who developed metastasis within one year. However, it is important to emphasize that the nomogram is currently intended for risk stratification and hypothesis generation rather than definitive treatment guidance, as no prospective validation or clinical impact analysis has been performed. For potential clinical application, LA-NPC patients with a predicted risk exceeding 8.7% are considered at high risk for metastasis within one year. Consequently, intensified treatment strategies, such as additional cycles of induction chemotherapy, adjuvant chemotherapy, or immunotherapy, could be considered. Furthermore, more frequent follow-up and advanced imaging diagnostics, such as PET-CT, might be considered in future research for these high-risk patients. The threshold can be adjusted to be more conservative or aggressive depending on the clinical context by selecting different cut-off points based on the PDF and CUC analyses. However, it is acknowledged that approximately 17% of patients who would not develop metastasis might receive unnecessary intensified treatment, and this proportion could be further optimized. Future research should investigate additional biomarkers to enhance the efficiency of this predictive model.

This study has several limitations. First, its retrospective design inevitably introduces selection bias. Second, detailed information on certain patient characteristics was limited due to the retrospective nature of the data collection. Third, all patients were recruited from a single center, which may introduce sample bias and limit the generalizability of the findings. Fourth, patients were treated between 2009 and 2012, and although the treatment paradigms have evolved with the introduction of newer chemotherapy regimens and immunotherapy, the model’s applicability to contemporary practice warrants external validation. The main strengths of this study include the relatively large sample size, the inclusion of an internal validation cohort, the incorporation of metabolic syndrome into the predictive model.

In conclusion, this study establishes the first nomogram to incorporate metabolic syndrome (MetS) for the specific prediction of metastasis within one year after radiotherapy in LA‑NPC patients. The developed nomogram demonstrates good predictive accuracy for estimating this risk. However, external validation using independent cohorts from other institutions is essential before clinical implementation. To standardize its clinical application, prospective validation studies are warranted.

## Data Availability

The data that support the findings of this study are available from the corresponding author(P.F.) upon reasonable request.
